# Supplementary material for: RNA-Seq Analysis Reveals an Essential Role of the cGMP-PKG-MAPK Pathways in Retinal Degeneration Caused by Cep250 Deficiency
Source: Int J Mol Sci. 2023 May 16;24(10):8843. doi: 10.3390/ijms24108843 (PMC10218315; doi:10.3390/ijms24108843)
Supplement: Supplementary file 1 [file ijms-24-08843-s001.zip › ijms-2368809-Supplementary Figures S1-S3.pdf]

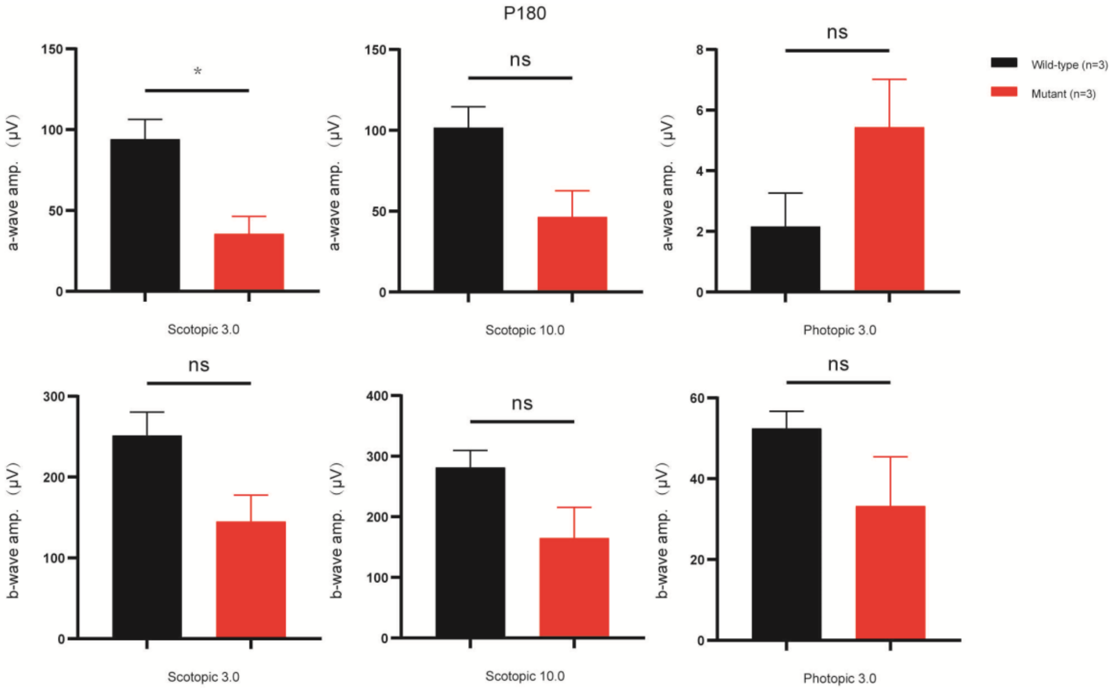

Supplementary Figure S1. Retinal function of Cep250 KO and WT mice at P180 determined by ERG. Statistical analysis of a-wave and b-wave amplitudes. *P < 0.05, ns = no significance. Student's t-test.

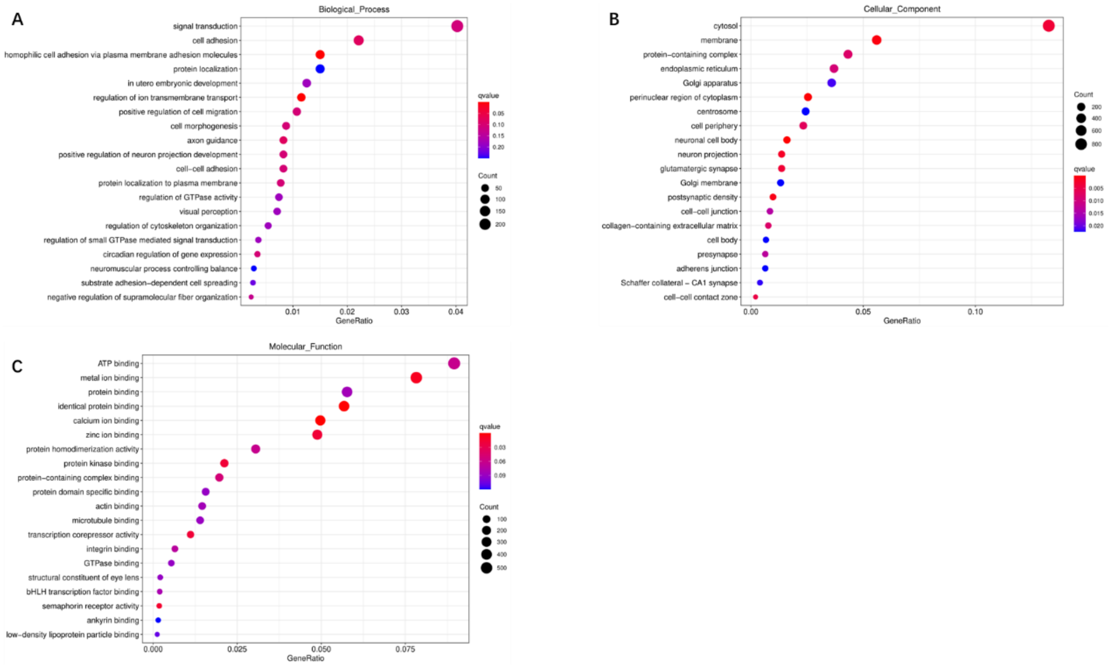

Supplementary Figure S2. GO enrichment analysis of the DEGs from Cep250 KO retinas compared to WT retina. (A) involved in Biological_Process (BP) of GO enrichment. (B) involved in Cellular_Component (CC) of GO enrichment. (C) involved in Molecular_Function (MF) of GO enrichment.

A

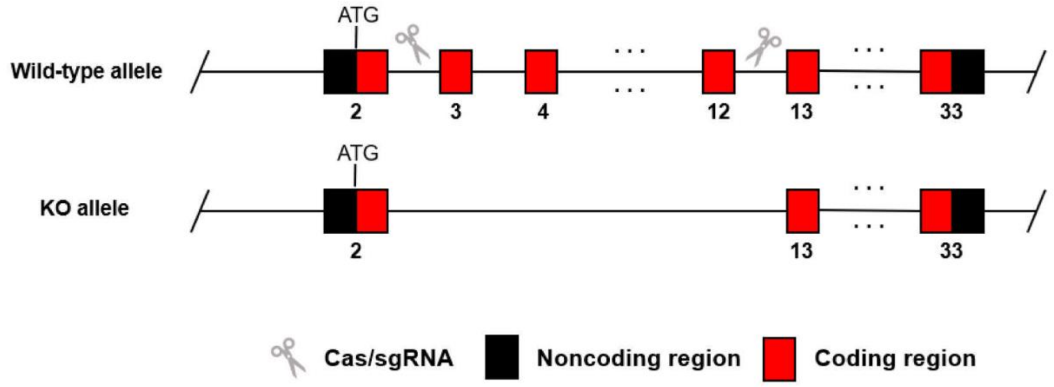

B

PCR number	Primer name	Primer sequences	PCR products
1. KO	Cep250-KO-F	CGTGTAGTACCAAACATCCTGGTG	WT:9380bp Targeted:~799bp
	Cep250-KO-R	AAGCCAAAATCTACAGCAACCCTC	
2. WT	Cep250-WT-F	CCCAACCTGGAACAGTTGCTGA	WT:438bp Targeted:0bp
	Cep250-WT-R	GCAAGGCTTTATTCACCACATCG	

C

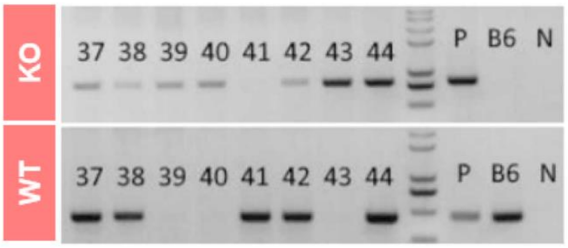

Genotyping results: Mutant: 39, 40, 43 Wild-type: 41

D

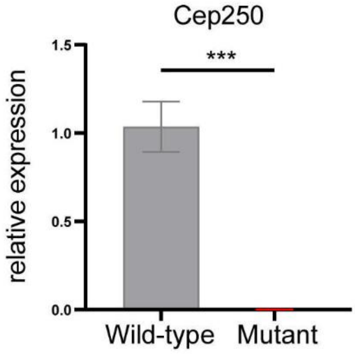

Supplementary Figure S3. Generation of Cep250 KO mice. (A) Cep250 KO mice were generated in a C57BL/6J background using the CRISPR/Cas9 system. (B-C) The Genotyping primers and example genotyping results of Cep250 KO mice and Wild-type mice. (D) RT-PCR analysis of Cep250 KO mice and Wild-type mice. ***P < 0.001, Student's t-test.
