# Supplementary material for: RNA-Seq Analysis Reveals an Essential Role of the cGMP-PKG-MAPK Pathways in Retinal Degeneration Caused by Cep250 Deficiency
Source: Int J Mol Sci. 2023 May 16;24(10):8843. doi: 10.3390/ijms24108843 (PMC10218315; doi:10.3390/ijms24108843)
Supplement: Supplementary file 1 [file ijms-24-08843-s001.zip › ijms-2368809-SupplementaryTable S1.pdf]

Table S1. Primer pairs used for qRT-PCR.

Gene	Primers
Cep250	5‘-GATGGCCGAGAATCAGGCTG-3’
	5‘-CCAGTGGCTTCTAGTCGCTT-3’
Pde3a	5‘-GGACTAGCGTGCTTAGGACG-3’
	5‘-GTATGCCAGGTAAGGTCTCCA-3’
Pde3b	5‘-AAAGCGCAGCCGGTTACTAT-3’
	5‘-CCATATTGCGAGCTTCATTTAGC-3’
Pde6b	5‘-GATCCAAGACAGTCCTCTCCAAG-3’
	5‘-GACCACACGAAAGGAGATAGTCA-3’
Parp1	5‘-GTGACTTTTTAGCGGAGTACGC-3’
	5‘-CCAGCGGTCAATCATACCCAG-3’
Fgf2	5‘-GCAAAAACGGGGGCTTCTTC-3’
	5‘-AACGGTTAGCACACACTCCT-3’
Edn2	5‘-GTGGTCGCCCCAAGCA-3’
	5‘-TCCACATCTTGCCAGTCTTTAACA-3’
Gfap	5‘-TCTCGAATGACTCCTCCACTC-3’
	5‘-AAGCTCCGCCTGGTAGACAT-3’
Tyr	5‘-CAAAGGGGTGGATGACCGTG-3’
	5‘-AACTTACAGTTTCCGCAGTTGA-3’
Gapdh	5‘-AGGTCGGTGTGAACGGATTTG-3’
	5‘-TGTAGACCATGTAGTTGAGGTCA-3’
